# Spin-wave propagation steered by electric field modulated exchange interaction

**DOI:** 10.1038/srep31783

**Published:** 2016-09-02

**Authors:** Sheng Wang, Xiawei Guan, Xiaomin Cheng, Chen Lian, Ting Huang, Xiangshui Miao

**Affiliations:** 1 School of Optical and Electronic Information, Huazhong University of Science and Technology, Wuhan 430074, China; 2 Wuhan National Laboratory for Optoelectronics, Wuhan 430074, China

## Abstract

Combined *ab initio* and micromagnetic simulations are carried out to demonstrate the feasibility on the electrical manipulation of spin-wave propagation in ultrathin Fe films. It is discovered that the exchange interaction can be substantially weakened under the influence of electric field applied perpendicular to the magnetic film surface. Furthermore, we demonstrate that the electric field modified exchange constant could effectively control the propagation of spin waves. To be specific, an external applied electric field of 5 V/nm can effectively weaken exchange interaction by 80% and is sufficient to induce nearly twofold change of the wavenumber. This discovery may open a door to energy-efficient local manipulation of the spin wave propagation utilizing electric fields, which is crucial for both fundamental research and spin wave based logic applications.

Magnonics is deemed as the most promising candidate for information transmission and processing^1^,^2^,^3^,^4^ where information is carried by the collective precession of the electrons’ spin (namely spin waves) instead of dissipative translation of their charge. Magnons (quanta of spin waves) allow for the transport and processing of spin information without the movement of any real particles, thus, it is free of Joule heat dissipation^5^,^6^. In magnonics, information can be encoded both in the amplitude^7^ and phase^8^, thereby the manipulation of spin-wave propagation lies at the core of promoting the practical application of magnonics^9^. Quite recently, remarkable progress has been made in the spin wave logic devices based on the phase^7^,^10^,^11^,^12^,^13^ as well as the amplitude^14^ of spin wave. However, so far, most of the implementations of logical functions rely on the generation of local Oersted fields to control the spin-wave dispersion relation to steer the spin wave propagation. Delicate as the designs are, there are some drawbacks and problems inherent to magnetic fields. First of all, generating local Oersted field through an underlying current-carrying stripe would complicate the device structure^14^ especially in curved waveguides, and the current-carrying wire will continuously draw power from the system which could inhibit the benefits of magnonic devices^1^. In addition, a spatial inhomogeneity in local fields and stray field from neighbouring branch will give rise to poor reliability and high bit error rates. Therefore, it would be of great importance to find a new method to manipulate the propagation of spin waves.

Benefited from the inspiration of CMOS technology where information is manipulated by electric fields with minimum power consumption, we present an energy-efficient local manipulation method for spin wave propagation utilizing electric-field-modulated exchange interaction in this article. The feasibility of electrical modulation on spin wave propagation makes CMOS-process-friendlier, lower-power-consumption and more compact magnonic devices possible in practical application.

Herein we carry out a multiscale and empirical parameter free calculation which combines *ab initio* and micromagnetic simulations^15^,^16^, to study the electrical modulation of spin wave propagation. Firstly, *ab initio* calculations are carried out to investigate the direct influence of an external electric field on exchange constant based on density function theory. It turns out that the external electric field would induce a notable change in the exchange stiffness of ultrathin Fe film. Then micromagnetic modelings of spin waves propagating in thin film under influence of electric fields are implemented with parameters obtained by *ab initio* calculations. Our results show that the external electric field can effectively control the propagation of spin waves.

## Methods and Results

First principles calculations based on density functional theory^17^,^18^,^19^ (DFT) were performed using the Vienna ab initio simulation package^20^,^21^ (VASP) to elucidate the magnetoelectric effect on the exchange constant quantitatively under the influence of a uniform electric field applied perpendicular to the film surface. The electronic exchange-correlation and electron-ion interaction were described within a generalized-gradient approximation (GGA) with the Perdew-Burke-Ernzerhof (PBE) functional^22^ and the projector augmented-wave (PAW) formalism^23^, respectively. The plane wave basis set with a cutoff kinetic energy of 500 eV, a 16 × 16 × 1 Monkhorst-Pack k-point mesh^24^ for Brillouin zone sampling and a self-consistent-field energy convergence criterion of 10^−7^ eV were employed^25^.

**Figure 1:**
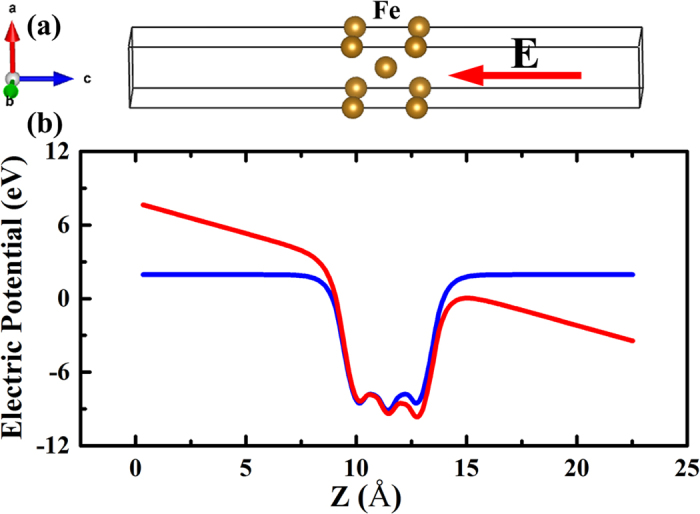
(**a**) Schematic view of the model which consists of 3-ML bcc Fe (arrows indicate the axes and direction of the electric field, respectively). (**b**) Planer-averaged electrostatic potential distribution alone the z axis at 0.0 (blue curve) and 5 V/nm (red curve) external applied electric field conditions.

Our general supercell under investigation involves a stack structure of 3 bcc Fe(001) (a = 2.866 Å, (ref. 26 )) monolayers (MLs) and a vacuum layer with a total thickness of 20 Å as shown in Fig. 1. The electric field along c axis was introduced by the dipole layer method^27^ with the dipole placed in the vacuum region of the supercell. The lower part of Fig. 1 illustrates the planer-averaged electrostatic potential distribution alone z axis under field-free and 5 V/nm external applied electric field conditions.

The configuration under study was fully relaxed along the c-axis without electric field until the residual force worked out to be less than 1 meV/Å. The exchange integral *J* was extracted from the total energy difference between antiferromagnetic (AFM) and ferromagnetic (FM) configurations.

To facilitate description, we label the three Fe atoms as site 1, 2 and 3 from left to right. For simplicity, only the nearest neighbor exchange interaction is taken into consideration. Thus we have the following expressions for the total ferromagnetic (E_F_) and antiferromagnetic (E_AF_) energies:


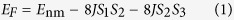



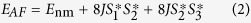


where *E*_*nm*_ is the nonmagnetic part of the total energy, *J* is the exchange integral, and *S*_*i*_ (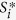) is the total spin of atom *i* in ferromagnetic (antiferromagnetic) configuration in unit of ℏ.

Combining equations (1)–(2), 2, the exchange integral can be calculated,


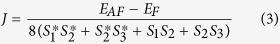


The phenomenological parameter exchange constant *A* can be related to the microscopic exchange integral in the Heisenberg model of ferromagnetism. For a body-centered cubic lattice, *A* is given by


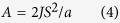


where *a* is the lattice constant and the exchange stiffness *D* is related to *J* by


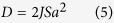


**Figure 2:**
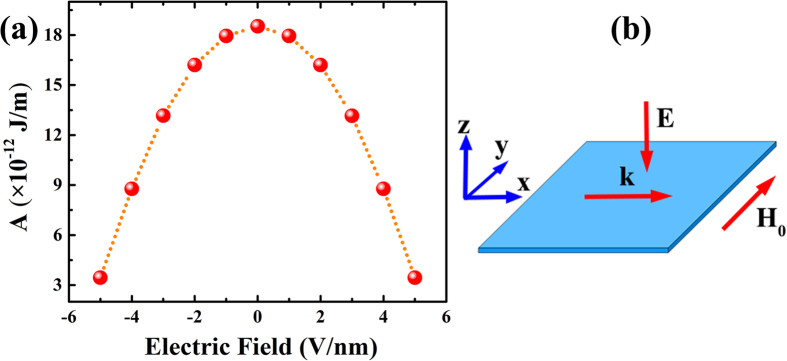
(**a**) Exchange constant *A* as a function of external electric field. (**b**) Excitation geometry for surface spin waves, in which both the DC bias magnetic field *H*_*0*_ and wave vector *k* are in the plane of thin film, but are mutually perpendicular to each other.

Results of our first principle calculations demonstrate that exchange constant do change under applied electric field, well in consistent with the experiment finding reported elsewhere^28^. Figure 2(a) shows the calculated exchange constant of 3-ML Fe (001) film as a function of an applied electric field perpendicular to film surface. It is seen that the exchange constant changes nonlinearly with the electric field in contrast to the well-known linear magnetoelectric effects such as electric field modulated magnetic anisotropy and saturation magnetization^29^. To be specific, the zero electric field exchange constant *A* is calculated to be 1.85 × 10^−11^J/m, well in agreement with the counterpart (1.88 μerg/cm) estimated from the Curie temperature of bcc Fe^30^. Detailed calculation results of the exchange integral *J*, exchange constant *A* and exchange stiffness *D* are listed in Table 1. As is shown in Fig. 2(a), the exchange constant decreases dramatically with the increase of applied electric field. In particular, when an electric field of 0.5 V/Å applied, a significant change of about 80% in the exchange constant can be observed. In addition, there is an enhancement in magnetic moment at the surface, originating from sudden change in the electronic structure of the atomic orbitals along the direction perpendicular to the surface as compared with the bulk due to the break of symmetry at the surface^31^.

In the latter part of this article, we proposed a novel electrical modulation technique of spin wave length and phase velocity, using object-oriented micromagnetic framework^32^ with variable exchange interactions by the application of electric fields,


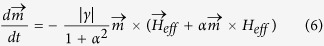


where 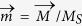 is the normalized magnetization vector with *M*_*S*_ the saturation magnetization, *α* is the Gilbert damping constant and *γ* is the Gilbert gyromagnetic ratio. 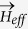 is the effective field, including Heisenberg exchange, magnetic anisotropy, demagnetization, and external applied magnetic fields.


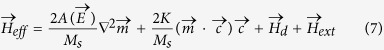


It is particularly pointed out that the exchange constant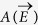 is electric-field-dependent as predicted above. Considering the individual moment and MAE contributions from the two surfaces are of opposite sign, the changes in moment and MAE at these two surfaces will compensate each other^33^. Therefore, we neglected the moment and anisotropy change at the surface caused by the electric-field in the micromagetic simulations. Here, we consider an ultrathin magnetic film with the dimension of 500 × 500 × 0.2866 nm^3^ in the numerical simulation. Parameters taken for simulation are: 1) saturation magnetization *M*_*S*_ = 1.75 × 10^6^ A/m; 2) Gilbert damping constant *α* = 0.03; 3) anisotropy constant *K* = 0 (here we neglect the relatively small magnetic anisotropy energy in cubic iron); 4) Gilbert gyromagnetic ratio *γ* = 2.211 × 10^5^m/(A·s); 5) DC bias field *μ*_0_*H*_*0*_ = 1 T. A stringent condition to follow in the creation of the meshes is that the maximum mesh element size should not exceed the exchange length of the material. The exchange length 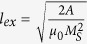 of ultrathin iron film under electric-field-free and a uniform 5 V/nm applied electrical field conditions are 3.1 nm and 1.3 nm, respectively. We set the mesh size to 1 nm × 1 nm × 0.2866 nm and apply a 2D periodic boundary condition.

The magnetization state for the configuration was first fully relaxed under the bias magnetic field along y direction. Then an excitation source varying as cosine function in time at a frequency of 4 × 10^11^ rad/s was applied to excite surface spin wave^34^ travelling alone x direction as shown in Fig. 2(b).

**Figure 3:**
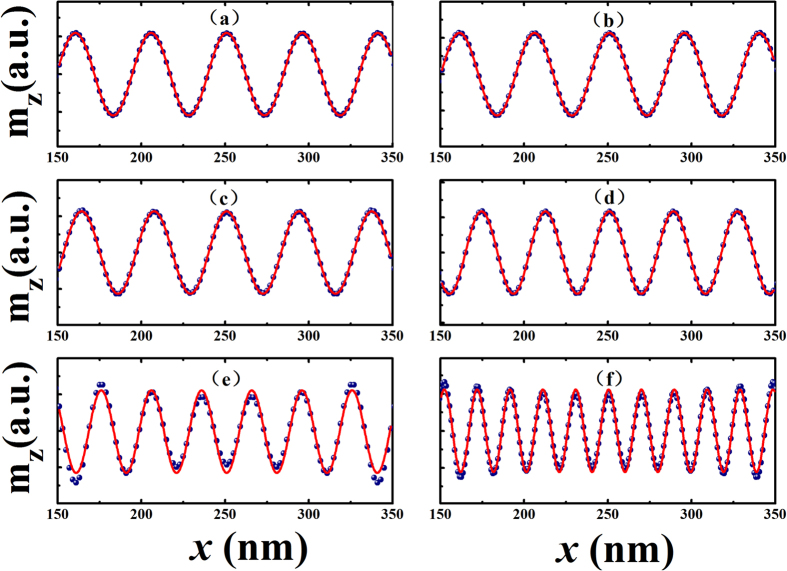
Spatial domain characterization of surface spin waves for case with (**a**) zero, (**b**) 1 V/nm, (**c**) 2 V/nm, (**d**) 3 V/nm, (**e**) 4 V/nm and (**f**) 5 V/nm applied electric field. A cosine excitation source with a frequency of 4 × 10^11^ rad/s has been applied for micromagnetic simulations and the solid lines in red are cosine fits to the calculated data.

The micromagnetic simulation results in Fig. 3(a–f) show the spatial domain characterization of surface spin waves with applied electric field normal to the film. It is clear that the wave number increases by enhancing the electric field from zero to 5 V/nm as illustrated in Fig. 3. In other words, the phase velocity and wavelength of surface spin wave can be changed via external electric field, which also applies to backward volume (BV) and forward volume (FV) waves. Therefore, the application of an electric field around 5 V/nm can decrease the wavelength by half as compared to the field-free one.

**Figure 4:**
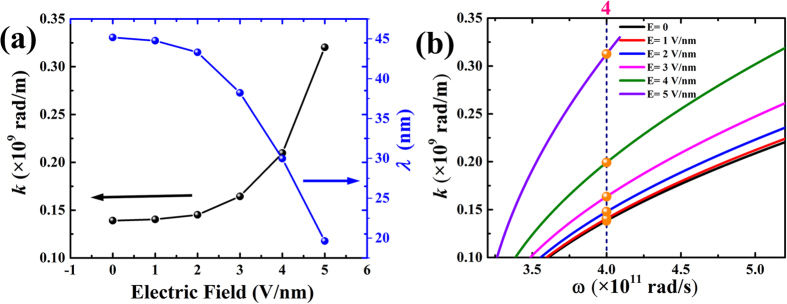
(**a**) Electric-field-induced changes in calculated surface spin wave wavenumber *k* and wavelength *λ*. (**b**) Theoretical dispersion relation curves for models used in simulation. Points at ω = 4 × 10^11^rad/s are highlighted as yellow balls. The x-y axes have been exchanged for comparison with micromagnetic simulation results in Fig. 4(a).

The electrical modulation of the wavelength can be interpreted by the spin wave dispersion relation. A useful analytic approximation for spin wave dispersion was derived by Kalinikos^35^, the analytical formula for spin wave dispersion in a finite-size ferromagnetic film after taking exchange interactions into account is given by^36^


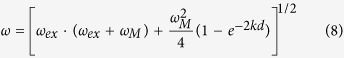
where *ω*_ex_ = *ω*_0_ + *λ*_ex_*ω*_*M*_*k*^2^, *ω*_0_ = *γμ*_0_*H*_0_, *ω*_*M*_ = *γμ*_0_*M*_*s*_, 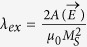 and *d* is the thickness of the thin film. Figure 4(b) reflects the theoretic dispersion relation based on equation (8) given the field-modulated exchange constant effect. As the strength of applied electric field increases, the exchange interaction is reduced, shifting the dispersion branch downwards in frequency in the *ω-k* view. For a given frequency of the spin wave this results in an upshift of the wavenumber *k*. It’s obvious that simulation result in Fig. 4(a) is in excellent agreement with that obtained from theory. Detailed results are provided in Table 2.

Utilizing the dependence of the carrier wave number of the spin-wave for a given carrier frequency on the external electric field applied perpendicular to the ferromagnetic film, it is easy to implement electric-field-controlled phase shifter. Our findings will throw some light on the technical feasibility of microscopic spin-wave logic devices based on the electric-field-controllable manipulation of the spin wave phase^7^,^37^. In contrast to previous scheme where the control current is “normally on” to maintain the control magnetic field, local manipulation method for spin wave propagation utilizing electric field is energy-efficient for the system is “normally off”. It rarely consumes power dissipation except for the charge/discharge period during which the control state switches.

## Conclusion

In conclusion, we have discovered the electric field control of exchange interaction in ultrathin magnetic film from first principle calculation and investigated its tuning effect on spin wave propagation by micromagnetic simulation. To be specific, an external applied electric field of 5 V/nm can effectively weaken exchange interaction by 80% and is sufficient to induce nearly twofold changes of the wavenumber. Therefore, this significant electric-field-modulation effect on the exchange constant is likely to pave another feasible route for the implementation of nanoscale spin-wave logic devices.

## Additional Information

**How to cite this article**: Wang, S. *et al*. Spin-wave propagation steered by electric field modulated exchange interaction. *Sci. Rep.* **6**, 31783; doi: 10.1038/srep31783 (2016).

## Figures and Tables

**Table 1:** The exchange integral *J*, exchange constant *A* and exchange stiffness *D* for 3-ML Fe under electric field ranging from zero to 5 V/nm.

Electric Field (V/nm)	*J* (meV)	*A* (×10^−12^ J/m)	*D* (meV·Å^2^)
0	9.985	18.520	225.432
±1	9.674	17.945	218.248
±2	8.757	16.204	196.934
±3	7.049	13.155	157.951
±4	4.666	8.776	103.659
±5	1.817	3.444	40.000

**Table 2:** The surface spin wave length *λ*, wavenumber *k* and wavenumber in theory *k*_*th*_ for models under electric field ranging from zero to 5 V/nm.

Electric field (V/nm)	*λ* (nm)	*k* (×10^9^ rad/m)	*k*_*th*_ (×10^9^ rad/m)
0	45.170	0.139	0.139
±1	44.768	0.140	0.141
±2	43.300	0.145	0.148
±3	38.217	0.164	0.164
±4	29.967	0.210	0.199
±5	19.621	0.320	0.313
